# Important Role of the GLP-1 Axis for Glucose Homeostasis after Bariatric Surgery

**DOI:** 10.1016/j.celrep.2019.01.047

**Published:** 2019-02-05

**Authors:** Pierre Larraufie, Geoffrey P. Roberts, Anne K. McGavigan, Richard G. Kay, Joyce Li, Andrew Leiter, Audrey Melvin, Emma K. Biggs, Peter Ravn, Kathleen Davy, David C. Hornigold, Giles S.H. Yeo, Richard H. Hardwick, Frank Reimann, Fiona M. Gribble

**Affiliations:** 1 Metabolic Research Laboratories, Wellcome Trust MRC Institute of Metabolic Science, Addenbrooke’s Hospital, Hills Road, Cambridge CB2 0QQ, UK; 2 Department of Medicine, University of Massachusetts Medical School, Worcester, MA, USA; 3 Department of Antibody Discovery and Protein Engineering, MedImmune, Granta Park, Cambridge CB21 6GH, UK; 4 Department of Cardiovascular and Metabolic Disease, MedImmune, Granta Park, Cambridge, UK; 5 Cambridge Oesophago-gastric Centre, Addenbrooke’s Hospital, Cambridge, UK

**Keywords:** bariatric surgery, GLP-1, enteroendocrine cells, peptidomics, mass spectrometry, transcriptomics, intestinal transit, gut hormones

## Abstract

Bariatric surgery is widely used to treat obesity and improves type 2 diabetes beyond expectations from the degree of weight loss. Elevated post-prandial concentrations of glucagon-like peptide 1 (GLP-1), peptide YY (PYY), and insulin are widely reported, but the importance of GLP-1 in post-bariatric physiology remains debated. Here, we show that GLP-1 is a major driver of insulin secretion after bariatric surgery, as demonstrated by blocking GLP-1 receptors (GLP1Rs) post-gastrectomy in lean humans using Exendin-9 or in mice using an anti-GLP1R antibody. Transcriptomics and peptidomics analyses revealed that human and mouse enteroendocrine cells were unaltered post-surgery; instead, we found that elevated plasma GLP-1 and PYY correlated with increased nutrient delivery to the distal gut in mice. We conclude that increased GLP-1 secretion after bariatric surgery arises from rapid nutrient delivery to the distal gut and is a key driver of enhanced insulin secretion.

## Highlights

• Increased postprandial GLP-1 triggers higher insulin levels after bariatric surgery
• Bariatric surgery does not change enteroendocrine cell identity or hormone content
• Increased nutrient flow to the distal gut after surgery enhances GLP-1 secretion

## Graphical Abstract

**Figure:**
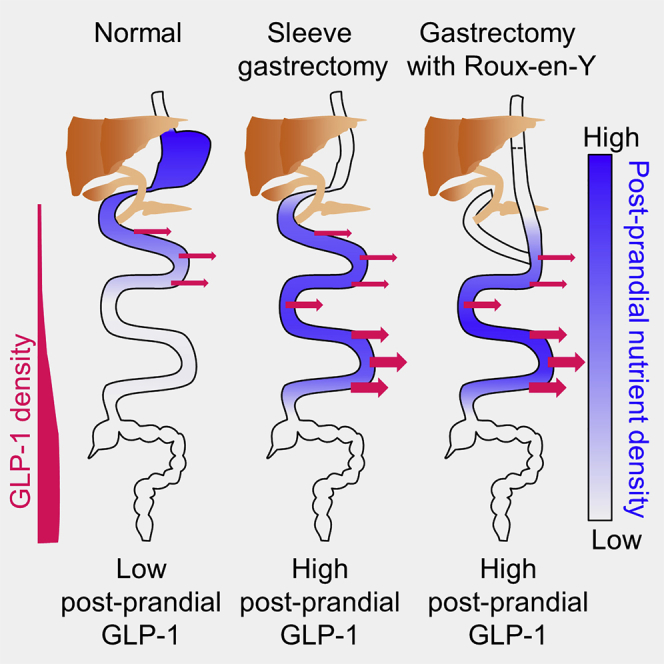

## Teaser

Bariatric surgery is associated with enhanced postprandial gut hormone release, particularly of GLP-1, which increases insulin secretion and glucose clearance. Larraufie et al. show that higher gut hormone levels are due not to changes in enteroendocrine cell characteristics or tissue hormone content but to altered flow of nutrients that stimulates more distal enteroendocrine cells.

## Introduction

Bariatric surgery is widely used to treat obesity and is particularly effective because it results in dramatic improvements in type 2 diabetes (Sjöström, 2013). Reduced plasma glucose after bariatric surgery can be attributed partly to loss of body weight and adiposity, which in turn improves insulin sensitivity (Sjöström, 2013). In addition, bariatric patients have elevated post-prandial insulin secretion, and there are increasing reports of bariatric surgery being used to treat type 2 diabetes in patients who are not severely obese (Pok and Lee, 2014), as well as of post-prandial hypoglycemia occurring years after surgery when increased insulin release occurs on a background of improved insulin sensitivity following loss of body weight (Salehi et al., 2018). Understanding the physiological basis for elevated post-prandial insulin secretion after bariatric surgery is therefore important both for preventing hypoglycemia in susceptible post-surgical populations and for developing new therapeutic strategies to treat type 2 diabetes.

We have studied the endocrinology of lean patients who underwent gastrectomy with Roux-en-Y gastric bypass (RYGB) reconstruction for the treatment or prophylaxis of gastric cancer (Roberts et al., 2018b). The surgical procedure is similar to a standard RYGB performed for obesity, with the exception that the stomach is removed in its entirety. Post-gastrectomy patients have elevated plasma glucagon-like peptide (GLP) 1, peptide YY (PYY), and insulin levels after an oral glucose tolerance test (OGTT), mirroring the endocrine changes seen in bariatric patients, but because these patients are not generally obese, the excessive insulin secretion is associated with significant rates of post-prandial hypoglycemia (Roberts et al., 2018b). The contribution of GLP-1 to the observed post-surgical changes in plasma glucose and insulin concentrations has been debated, as detailed in several reviews (Hutch and Sandoval, 2017, Smith et al., 2018). In obese post-bariatric patients, blocking GLP-1 action using Exendin-9 reduced insulin secretion and the incidence of hypoglycemia (Jørgensen et al., 2013, Salehi et al., 2014), but corresponding data from mouse models have been conflicting. Mice with global GLP-1 receptor (*Glp1r*) knockout, for example, exhibited similar weight loss and glucose tolerance to wild-type controls after vertical sleeve gastrectomy (VSG) (Wilson-Pérez et al., 2013), whereas another group reported that mice with inducible β cell-specific *Glp1r* knockout had impaired insulin secretion and higher plasma glucose after VSG (Garibay et al., 2016).

Why post-prandial GLP-1 and PYY levels are elevated after bariatric surgery remains incompletely elucidated. GLP-1 and PYY are produced from enteroendocrine cells (EECs), which comprise ∼1% of the intestinal epithelium (Gribble and Reimann, 2016). These cell types have been extensively characterized in mice, because they can be tagged with fluorescent reporters driven by cell-specific hormonal or transcription factor promoters in transgenic mouse models (Gribble and Reimann, 2016), but data on human EECs are limited, because cell purification requires antibody staining for identification (Roberts et al., 2018a). One potential explanation for the post-surgical changes in gut hormone release is that EECs undergo adaptive changes, producing more GLP-1 and PYY that can be mobilized after food intake, or changing their response to nutrients due to different receptor expression. Although immunostaining of intestinal biopsies from bariatric patients and obese rodent models does not support the concept that major shifts occur in the numbers of EECs producing different gut hormones (Mumphrey et al., 2013, Rhee et al., 2015), staining methods are semiquantitative at best and do not inform on receptor expression. However, an important role for intestinal adaptation was not supported by the finding that GLP-1 levels after gastric bypass surgery were higher when a liquid meal was delivered via the oral route than it was when delivered via the gastroduodenal route on consecutive days (Dirksen et al., 2010). An alternative explanation is that ingested nutrients make contact with and thereby stimulate more EECs from the distal gut after surgery, due to anatomic intestinal rearrangement and/or increased intestinal transit. In both humans and mice, GLP-1 and PYY production is higher in more distal regions of the small intestine (Roberts et al., 2018a), so increased distal nutrient delivery has the potential to activate a greater number of GLP-1 and PYY-producing EECs.

The objectives of this study were to explore the importance of GLP-1 in post-bariatric physiology and the mechanisms underlying elevated post-prandial GLP-1 secretion in this group. Studies were performed in lean human and mouse models to reduce the confounding effects of metabolic changes due to loss of body weight and adiposity.

## Results

### Role of GLP-1 in Driving Hyperinsulinemia in Humans

**Figure 1:**
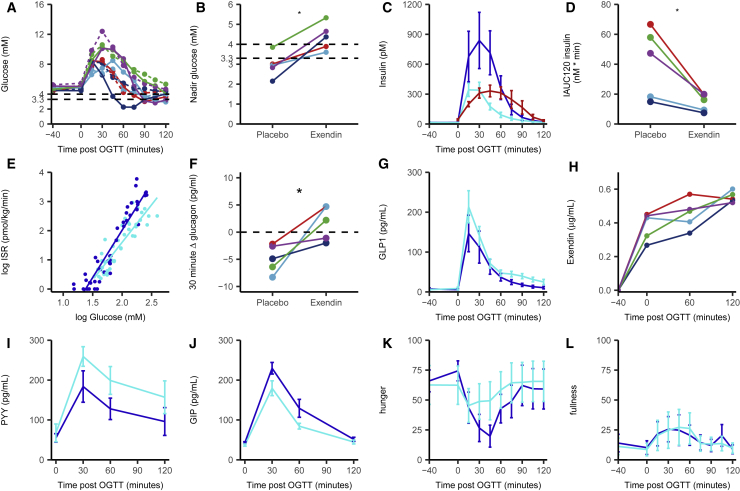
Exendin-9 Infusion in Post-gastrectomy Participants Receiving a 50 g OGTT Plasma parameters from 5 post-gastrectomy participants receiving either Exendin-9 or placebo in a cross-over design and challenged with a 50 g OGTT at time = 0. (A) Plasma glucose levels on placebo infusion (solid lines) or Exendin-9 infusion (dotted lines). Colors indicate individual participants. (B) Nadir glucose concentrations, taken from data shown in (A). (C) Plasma insulin concentrations for gastrectomy patients given placebo (light blue) or Exendin-9 (dark blue) or control patients (red, control data from previous study) (Roberts et al., 2018b). (D) Incremental area under the curve of insulin levels over 120 min. Colors represent individuals. (E) Correlation between log insulin secretion rate (ISR) and log glucose concentration using all measured time points after the OGTT during placebo (dark blue) or Exendin-9 (light blue) infusion. (F) Delta plasma glucagon concentrations between 0 and 30 min after the OGTT in either placebo- or Exendin-9-infused post-gastrectomy patients. Individuals are paired. (G–L) Total GLP-1 (G), PYY (I), and GIP (J) concentrations and hunger (K) and fullness (L) ratings in placebo-infused (dark blue) or Exendin-9-infused (light blue) gastrectomy patients. Data are represented as mean ± SD. Areas under the curve between placebo and Exendin-9 are statistically different for GLP-1, PYY, and GIP, with p < 0.05 using paired Student’s t test. (H) Exendin-9 concentrations measured during the Exendin-9 infusion. Colors represent individuals. ^∗^ indicates that the two groups are statistically different with p < 0.05 using paired Student’s t test.

We hypothesized that elevated plasma GLP-1 levels triggered by glucose ingestion were responsible for the high insulin secretion rates and subsequent hypoglycemia observed in our lean human cohort after gastrectomy (Roberts et al., 2018b), as reported previously in bariatric patients (Craig et al., 2017, Jørgensen et al., 2013, Salehi et al., 2014). Five post-gastrectomy patients were enrolled into a randomized, double-blind, placebo-controlled cross-over study, in which they received infusions of the GLP1R antagonist Exendin-9 or placebo on separate visits. Forty minutes after starting the infusion, they consumed a 50 g glucose drink, and 125 min later, they had an *ad libitum* test meal. Nadir glucose concentrations after the OGTT increased significantly from the control to the Exendin-9 day (Figures 1A and 1B). Elevated insulin concentrations were seen in the control arm and were significantly blunted by Exendin-9, reaching levels similar to those measured previously in a non-surgical control group (Roberts et al., 2018b) (Figures 1C and 1D). The inhibitory effect of Exendin-9 on insulin release was also observed as a reduced slope of the insulin secretory rate versus glucose concentration graph (Figure 1E). Glucagon concentrations 30 min after the OGTT were increased by Exendin-9 (Figure 1F), consistent with the known inhibitory effect of GLP-1 on glucagon secretion (Nauck et al., 1993). GLP-1 concentrations were higher with Exendin-9 (Figure 1G), consistent with previous reports that GLP-1 inhibits its own secretion (likely indirectly, e.g., via local somatostatin release) (Hansen et al., 2000, Heruc et al., 2014, Sze et al., 2011). Steady-state Exendin-9 concentrations (Figure 1H) were ∼0.4 μg/mL (∼120 nmol/L), ∼2-fold above the binding affinity of Exendin-9 for GLP1R in human insulinoma cells (Waser and Reubi, 2011). PYY concentrations were higher after Exendin-9 than after placebo (Figure 1I), mirroring the elevated GLP-1 levels and likely reflecting that PYY and GLP-1 are released from the same EEC type (Billing et al., 2018, Habib et al., 2013). Glucose-dependent insulinotropic polypeptide (GIP) concentrations were reduced by Exendin-9 (Figure 1J), suggesting that endogenous GLP-1 enhances GIP secretion—a finding not previously reported. Hunger scores were less suppressed by glucose ingestion in the Exendin-9 than in the placebo arm, without corresponding changes in fullness, suggesting that elevated GLP-1 concentrations contribute to reduced sensations of hunger in this cohort (Figures 1K and 1L).

### Role of GLP-1 in a Murine Lean VSG Model

**Figure 2:**
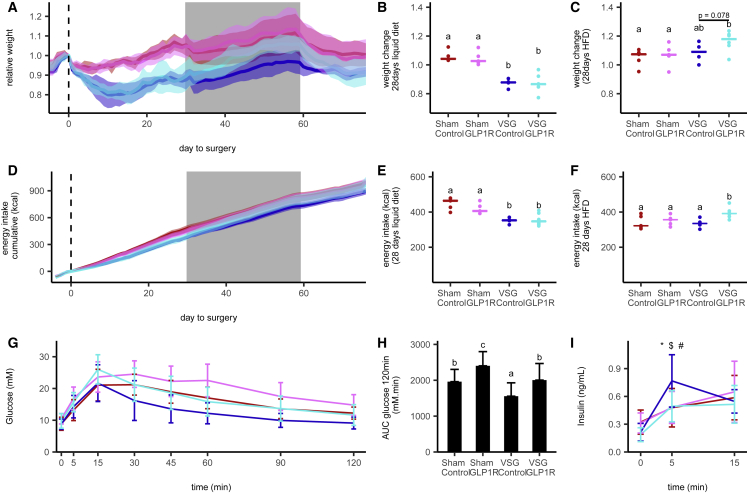
GLP1R Blockade in VSG-Operated Mice (A and D) Weight relative to surgery day (A) and cumulative energy intake (D) over time of vertical sleeve gastrectomy (VSG)-operated mice treated with control antibody (n = 4, dark blue) or GLP1R antibody (n = 6, light blue) and sham-operated mice treated with control antibody (n = 5, dark red) or GLP1R antibody (n = 5, pink). Data are mean ± SD. (B and E) Weight change (B) and cumulative energy intake (E) relative to the surgery day after 28 days of liquid diet. (C and F) Weight change (C) and cumulative energy intake (F) relative to the day of diet change from liquid to high fat after 28 days on high-fat diet. Data are median and individual values; significant differences between groups are assessed by Kruskal-Wallis followed by Dunn’s test; groups differing significantly with p < 0.05 are indicated by different letters. (G and I) Plasma glucose (G) and insulin (I) levels over time in response to a 1 g/kg OGTT in VSG- and sham-operated mice treated with control or GLP1R antibody. (H) Glucose area under the curve over 120 min for data as in (G). Colors in (B)–(I) are as described in (A). Data in (G)–(I) are the mean ± SD across animals and experiments of three OGTTs done 2, 4, and 10 weeks after surgery. ^∗^, $, and # indicate a difference with p < 0.05 between the VSG control antibody group and the VSG GLP1R-Ab, sham control-Ab, and sham GLP1R-Ab, respectively. Statistical differences between groups for the OGTT samples were assessed using a linear mixed model taking into account the repeated measures.

We established a model of gastrectomy in lean mice, in which animals had either a VSG or a sham control operation (McGavigan et al., 2017) (Figure S1). As expected, the VSG group lost more weight during the first week after surgery than the sham controls, associated with reduced food intake. OGTTs triggered higher plasma GLP-1 and insulin levels and lower glucose excursions in VSG than in sham control groups. This lean VSG model was used to examine the effect of an antagonistic anti-GLP1R antibody, providing long-lasting blockade of GLP1R (Biggs et al., 2018). Mice were injected weekly with anti-GLP1R or isotype control antibody for 12 weeks, beginning 1 day before VSG or sham surgery. After surgery, all groups received 4 weeks of liquid diet, followed by 4 weeks of high-fat diet (HFD) and then 12 days of the control low-fat diet (LFD), to assess whether the response to surgery was diet dependent. Peak and trough antibody titers are shown in Figure S2A. Weight loss in the post-operative period was higher in VSG than in sham mice, and liquid food intake was correspondingly reduced, but no differences were observed between the control and the active antibody groups (Figures 2A, 2B, 2D, and 2E). When transferred to HFD, by contrast, the VSG group on anti-GLP1R antibody (Ab) paradoxically ate significantly more than VSG mice given isotype control and showed a trend toward additional weight gain (p = 0.08 versus VSG controls) (Figures 2A, 2B, 2C, and 2F), suggesting that endogenous GLP-1 suppressed intake of HFD despite having little effect on ingestion of the liquid diet. OGTTs (1 g/kg) were performed one day after antibody injection at weeks 2, 4, and 10 after surgery (Figures 2G–2I; Figures S2C–S2K). Post-GTT plasma glucose concentrations were higher in mice given GLP1R than control antibody in both sham and VSG groups. Corresponding 5 min insulin levels were reduced in the anti-GLP1R-Ab VSG group compared with VSG isotype controls, whereas the anti-GLP1R antibody did not affect insulin levels in the sham group.

### Effect of Gastrectomy on the Intestinal Enteroendocrine Peptidome in Humans and Mice

**Figure 3:**
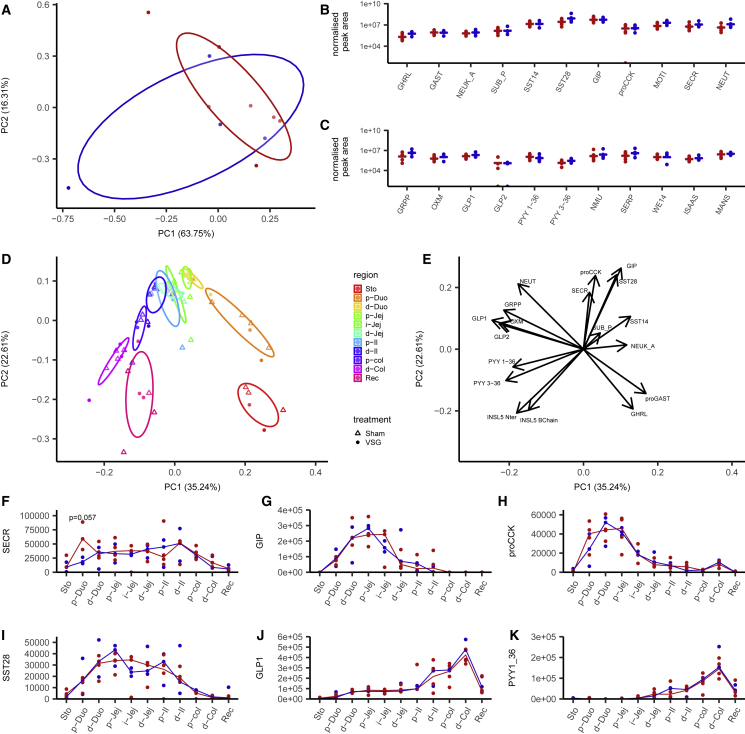
Effect of Bariatric Surgery on Tissue Peptide Content (A–C) Human jejunal peptidomics. (A) Principal-component analysis of the peptide content of human jejunal biopsies from patients before (n = 7, red) and after (n = 4, blue) gastrectomy surgery. Individual samples are plotted on the first two components representing all peptides measured in (B) and (C). (B and C) Peptide quantification for gut hormone peptides and granin-derived peptides for individual samples taken during (red) or after (blue) surgery. Data are normalized by tissue weight and internal standard for individual samples, and the medians are indicated. (D–K) Mouse peptidomics. (D and E) PCA of intestinal peptides measured in 3 VSG and 4 sham-operated mice in the stomach and every 5 cm along the gastrointestinal (GI) tract. Individual samples are color coded for their region of origin, and shape indicates the surgery type (D). Eigen vectors of each quantified peptide on the first two principal components (E). (F–K) Quantification of secretin (F), GIP (G), the N-terminal part of proCCK (H), SST28 (I), GLP-1 (J), and PYY1-36 (K) along the different regions of the GI tract, represented as median and individual samples from sham-operated (red) and VSG-operated (blue) mice. Differences between groups were assessed in each tissue for each peptide using a Mann-Whitney U test. Sto, stomach; Duo: duodenum; Jej, jejunum; Il, ileum; Col, colon; Rec, rectum; p, proximal; i, intermediate; d, distal; GHRL, ghrelin; proGAST, N terminus of proGastrin; NEUK_A, neurokinin A; SUB_P, substance P; SST14/28, somatostatin 14/28; GIP, glucose-dependent insulinotropic polypeptide; proCCK, N-terminal part of proCCK; SECR, secretin; NEUT, neurotensin; GRPP, glicentin-related peptide; OXM, oxyntomodulin; GLP1/2, glucagon-like peptide 1/2; PYY1-36/3-36, peptide YY1-36/3-36; INSL5 Nter, N-terminal part of INSL5 C-chain; INSL5 B-chain, B-chain of INSL5 (after reduction alkylation).

We investigated whether altered plasma gut hormone profiles observed after gastrectomy can be explained by changes in peptide biosynthesis in the gut. In lean human gastrectomy patients, we compared biopsies taken from the jejunum at the time of surgery, with biopsies taken by endoscopy after surgery from the same anatomic region, just distal to the site of anastomosis with the esophagus. Biopsies were examined by liquid chromatography-tandem mass spectrometry (LC-MS/MS), enabling the identification and quantification of 22 candidate secretory peptides. Principal-component analysis (PCA) of the samples across all measured peptides did not differentiate pre- and post-operative samples (Figure 3A), and no differences were detected when individual peptides were examined by multi-factorial ANOVA (Figures 3B and 3C). From the proglucagon peptide, we detected GLP-1(7-36amide), GLP-2, glicentin-related peptide (GRPP), and oxyntomodulin (OXM), but not pancreatic-type glucagon. GLP-1(7-36amide), PYY1-36, and PYY3-36 were not increased in the post-surgical intestinal biopsies, despite the raised plasma levels of total immunoreactive GLP-1 and PYY detected after an OGTT in post-gastrectomy patients.

Because it was not possible to examine other regions of the post-surgical human gut, we performed a similar study in mice after VSG or sham surgery. Samples were taken from the stomach to the rectum and analyzed by LC-MS/MS, as they were for the human jejunum. By PCA, we observed longitudinal gradients in hormone production in both sham and VSG mice but no substantial differences between the surgical groups (Figures 3D and 3E). The similarity between sham and VSG samples was also evident at the level of individual peptide profiles (Figures 3F–3K).

In the cohort of VSG and sham mice treated with GLP1R or control antibodies, we performed a similar LC-MS/MS analysis of pancreatic homogenates at the end of the protocol (samples taken ∼7 min after an Ensure gavage meal, as described later). Pancreatic levels of peptides derived from insulin, GCG, IAPP (islet amyloid polypeptide), PPY (pancreatic polypeptide), and PYY were similar across all groups; from the proglucagon peptide, we detected glucagon, GRPP, OXM, and GLP-1(1-37), but not GLP-1(7-37) or GLP-1(7-36amide) (Figures S3G–S3I).

### Effect of Gastrectomy on the EEC Transcriptome in Humans and Mice

**Figure 4:**
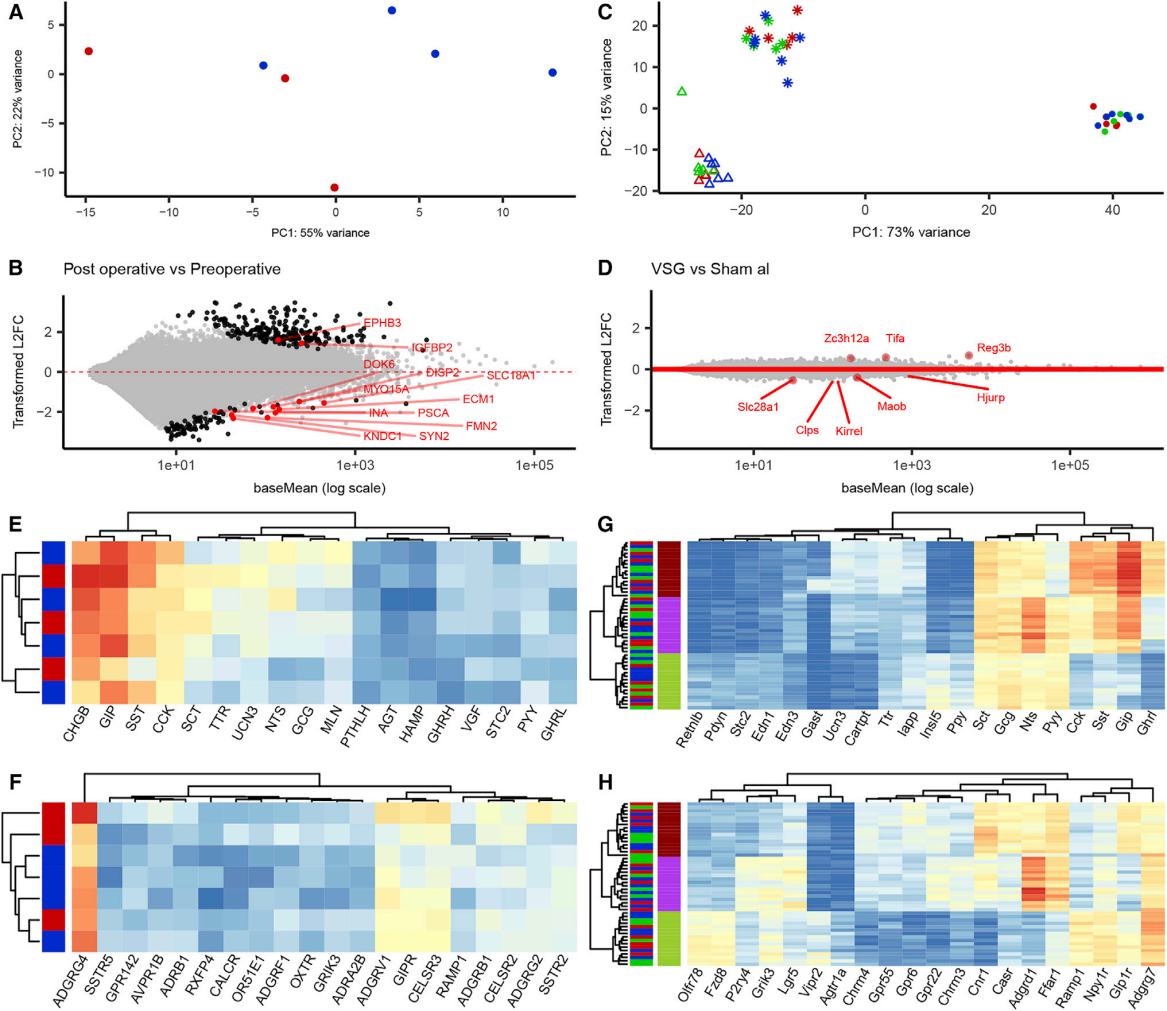
Transcriptomics of Human and Murine EECs after Gastrectomy Surgery (A) PCA of the 200 most variable genes significantly enriched in human jejunal EEC cells. Dots represent individual samples, color coded for before (red) or after (blue) gastrectomy and plotted on the first 2 principal components. (B) MA plot of post- versus pre-operative human samples representing, for each gene, the estimated log2 fold change between condition and mean normalized expression using a DESeq2 model. Genes that are differently expressed (padj < 0.05) are in black, and the differently expressed genes enriched in EECs are annotated in red. (C) PCA of the 500 most variable genes in murine EECs. Dots represent individual samples, color coded for sham *ad libitum* (red), sham weight-matched (green), and VSG-operated (blue) mice and shape coded for the tissue of origin on the first two principal components (Δ, top 5 cm of small intestine; ^∗^, bottom 15 cm of the small intestine; @, colon and rectum). (D) MA plot of VSG versus sham *ad libitum* samples representing the estimated log2 fold change between conditions across all 3 regions and the mean normalized expression of each gene using the DESeq2 model with interaction between surgery groups and regions. Genes that are differently expressed are annotated in red (adjusted p value [padj] < 0.05). (E–H) Heatmaps representing log2 normalized expression of the top variable EEC-enriched genes annotated as encoding hormones (E and G) or GPCRs (F and H) in human (E and F) and murine (G and H) samples. Samples and genes are clustered by Euclidean distance without scaling.

We next investigated whether the increased plasma GLP-1 and PYY levels after surgery were associated with transcriptomic adaptations in intestinal EECs, which could potentially change their responsiveness to food ingestion. In humans, we fluorescence-activated cell sorting (FACS)-purified EECs from the jejunum of peri-operative patients (collected during gastrectomy surgery) and from the same anatomic site in post-operative patients (collected by endoscopy) and performed transcriptomic analysis by RNA sequencing. EEC purity estimated from the FACS profiles ranged from 10% to 50%, compared with ∼0.1% in the starting cell populations, so to prevent bias introduced from non-EECs, we restricted the analysis to genes known to be differentially expressed and enriched in human EECs (Roberts et al., 2018a). By PCA, the pre- and post-surgical samples did not show any distinct clustering (Figure 4A), and the relatively few EEC genes that did exhibit significant differential expression between pre- and post-operative samples (Figure 4B) were not suggestive of major functional differences between the groups. Interrogation of the dataset for expression patterns of peptides, G-protein coupled receptors (GPCRs) (Figures 4E and 4F), transcription factors, and ion channels (Figures S4K and S4L) also revealed no segregation between pre- and post-operative groups.

We performed a similar study in the mouse VSG model using lean NeuroD1-cre/YFP mice (Li et al., 2012) fed chow diet after surgery. FACS-purified NeuroD1-positive cells from the top 5 cm or bottom 15 cm of the small intestine and the combined colon and rectum were analyzed from VSG, sham, and weight-matched sham animals by RNA sequencing. No differences in the percentages of sorted EECs were found among the groups (Figure S4B). PCA of the top 500 differentially expressed genes revealed that EECs differed according to the intestinal site from which they were collected, but not by treatment group (Figures 4C and 4D). The top 25 variable genes across all samples annotated as hormones, transcription factors, GPCRs, and ion channels are represented as heatmaps in Figures 4G, 4H, S4I, and S4J, revealing that genes involved in EEC function were distinct among different intestinal regions but not altered by surgery. Further clustering analyses examining samples from each region separately also did not reveal clustering by treatment based on the top 100 variable genes (Figures S4C–S4H).

### Altered Intestinal Transit after VSG in Mice

**Figure 5:**
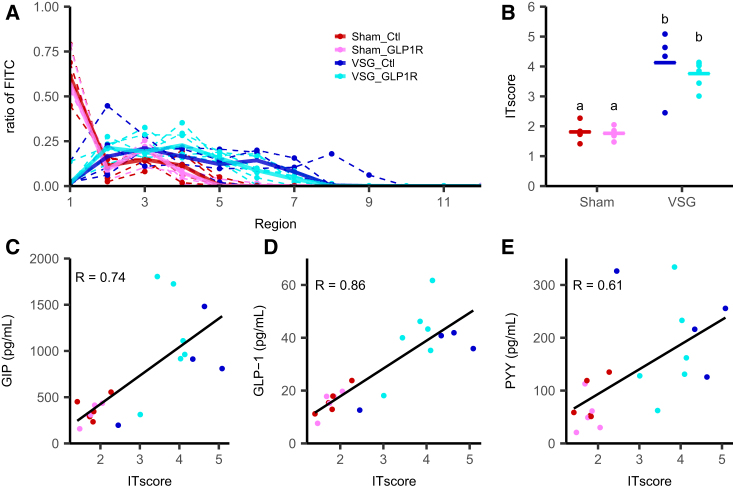
Intestinal Transit in VSG-Operated Mice (A) Ratio of fluorescence in each region of the GI tract (1, stomach; 2–9, small intestine (proximal to distal); 10, caecum; 11 and 12 colon and rectum) harvested 7 min after gavage. Dotted lines are individual mice, and solid lines are the median of each group (VSG-control antibody: blue, n = 4; VSG-GLP1R antibody: cyan, n = 6; sham-control antibody: red, n = 5; sham-GLP1R antibody: pink, n = 5). (B) Intestinal transit (IT) score measured as the weighted mean of the relative fluorescence by the tissue number. Data are individual and median. Significance between groups was assessed by Dunn’s test; groups differing significantly with p < 0.05 are indicated by different letters. (C–E) Correlation between plasma GIP (C), total GLP-1 (D), and total PYY levels (E) and intestinal transit score 7 min after gavage with 100 μL of Ensure plus with 0.5 mg FITC-Dextran 70kDa. Correlation coefficient was calculated using the Pearson correlation and all samples.

An alternative explanation for the increased plasma GLP-1 and PYY concentrations seen following an OGTT in humans and mice after gastrectomy is that ingested nutrients transit more quickly through the upper gut after surgery and penetrate to more distal regions of the gut before they are absorbed, thus targeting a larger and more distal pool of EECs. To test this hypothesis in the VSG model, mice were gavaged with a mixture of Ensure and fluorescein isothiocyanate (FITC) dextran and killed ∼7 min later to coincide with peak GLP-1 levels measured after OGTT for collection of the luminal contents, intestinal tissue, and plasma. The FITC contents of sequential segments of the stomach and intestines were measured by fluorescence, revealing that FITC dextran penetrated farther down the gut in VSG compared with sham mice, represented by a higher intestinal transit (IT) score (Figures 5A and 5B). The study was performed as a terminal step in the mice that had received either GLP1R antibody or isotype control for 12 weeks, but no difference in intestinal transit score was observed between those treated with the active and those treated with the control antibody. Across sham and VSG mice, plasma GLP-1, PYY, and GIP levels triggered by the Ensure liquid meal correlated with the intestinal transit score (Figures 5C–5E).

## Discussion

Lean patients who have undergone gastrectomy with RYGB, and mice after VSG, provide models to assess the metabolic responses to intestinal rearrangements without the confounding effects of profound weight loss seen in obese bariatric patients. Gastrectomy patients exhibit very high GLP-1, PYY, and insulin levels after an OGTT, mimicking the enteroendocrine physiology of bariatric surgery (Miholic et al., 1991, Roberts et al., 2018b).

The results of the Exendin-9 infusion in humans and anti-GLP1R antibody administration in mice demonstrate that elevated GLP-1 levels after glucose ingestion in these surgical groups are a strong driver of hyperinsulinemia. Peak insulin concentrations in gastrectomy patients were approximately 2-fold higher than in control subjects and were restored to normal levels by Exendin-9. Although other studies in obese humans have similarly concluded that GLP-1 plays an important role in driving insulin secretion after bariatric surgery (Jørgensen et al., 2013, Salehi et al., 2014), studies in mice have yielded conflicting results, with some groups arguing in favor (Garibay et al., 2016) and others against (Douros et al., 2018, Wilson-Pérez et al., 2013) this idea (Hutch and Sandoval, 2017, Smith et al., 2018). Additional improved glucose tolerance arising from concomitant weight loss and improved insulin sensitivity makes the interpretation of these types of study in mice particularly challenging. In practice, the elevated insulin secretion after bariatric surgery likely arises because of the combined rapid elevations of plasma glucose and GLP-1 occurring after glucose ingestion. Glucose concentrations rise faster after gastrectomy because the absence of a gastric reservoir results in rapid entry of ingested glucose into the small intestine, where it is absorbed into the bloodstream (Jacobsen et al., 2013). While pancreatic β cells generate a small secretory response to the rising plasma glucose concentration, this is strongly potentiated by GLP-1 (Gromada et al., 1998). After surgery, this synergy results in hyperinsulin secretion that is inappropriate for the ingested glucose load, and in lean gastrectomy patients with normal insulin sensitivity, the excessive insulin response can be sufficient to drive hypoglycemia. In obese diabetic bariatric patients who are relatively insulin resistant, the response is more likely to be seen as a beneficial lowering of already-elevated plasma glucose concentrations (Jørgensen et al., 2013).

Altered patterns of food intake are commonly reported after bariatric surgery, although the mechanisms remain incompletely explained (Kittrell et al., 2018, Mathes and Spector, 2012, Miras et al., 2012). The lean post-gastrectomy patients also report difficulties in maintaining body weight, although it is unclear whether this arises from alterations in the normal signals that control food intake or a learnt response resulting from the experience of post-prandial symptoms, commonly referred to as dumping syndrome. In the current study, we found that Exendin-9 increased hunger scores after the OGTT, suggesting that high GLP-1 levels contribute to the suppression of hunger in gastrectomy patients. Although elevated endogenous GLP-1 levels after bariatric surgery have not previously been linked to reductions in hunger, GLP-1 mimetics have been licensed as anti-obesity agents because of their suppressive effect on food intake (Nauck and Meier, 2018). The contribution of concomitantly raised PYY levels remains to be established, but in bariatric patients, PYY(3-36) was found to contribute to reduced food intake (Svane et al., 2016). Our finding that GLP1R blockade did not influence weight loss in the immediate post-operative period in mice is consistent with other reports that GLP-1 activity is not required for weight loss in this model (Wilson-Pérez et al., 2013, Ye et al., 2014). However, we did observe a significant increase in consumption of HFD in VSG mice after GLP1R blockade, suggesting that elevated GLP-1 might play a role in the suppression of high-fat consumption after surgery.

We found no evidence to support the hypothesis that EECs exhibit altered peptide production or stimulus responsiveness after surgery that could potentially explain the elevated GLP-1 and PYY concentrations triggered by food ingestion in these patients. It was surprising to find that when the jejunum is directly anastomosed to the esophagus in the human gastrectomy group, resulting in increased exposure to ingested nutrients and a loss of exposure to biliary secretions, there was no indication that its resident EECs altered their properties with respect to peptide production or general transcriptome. A similar lack of EEC adaptation was found along the length of the mouse gut, when we examined different regions by peptidomics and transcriptomics in the murine VSG model. Using LC-MS/MS, we did not detect pancreatic-type glucagon in the human or mouse intestine, or GLP-1 in mouse pancreas, either before or after surgery, despite reports that the gut can produce glucagon and the pancreas can produce GLP-1 under certain pathophysiological conditions (Chambers et al., 2017, Donath and Burcelin, 2013, Knop, 2018). The differences between our findings and those of others might reflect that we used LC-MS/MS to identify intact peptides, preventing false-positive detection caused by immunoassay cross-reactivity, or that alternative proglucagon processing occurs under stressful or pathological conditions such as obesity and diabetes, which were not present in our lean models.

When we measured intestinal transit and plasma gut hormone levels at a fixed time point after gavage feeding in mice, we found that that food passed considerably farther down the gut after VSG and that plasma GLP-1, PYY, and GIP concentrations were strongly correlated with the length of intestine exposed to the ingested nutrients. The likely explanation for this correlation is that enhanced gut hormone secretion after bariatric surgery arises when nutrients make contact with more EECs. GLP-1 and PYY concentrations exhibit particularly marked elevations after surgery, because these hormones are produced at higher levels in distal than proximal EECs, and whereas readily digestible nutrients are normally absorbed high up in the gut so that they make little contact with these distal EECs, a shift in nutrient absorption to the lower gut targets a larger GLP-1 and PYY-producing cell population.

To conclude, the results presented here suggest that the following sequence of events occurs after bariatric and gastrectomy procedures, with substantial impacts on metabolism. The anatomic rearrangements and altered nutrient flow and digestion do not alter EEC properties, but increased nutrient transit to the distal gut results in enhanced exposure of distal EECs to ingested stimuli and consequent elevations of GLP-1 and PYY secretion. GLP-1, despite having some effect on hunger, is not required for early post-operative weight loss, but it is a strong driver of insulin secretion, acting in synergy with high glucose peaks resulting from rapid glucose absorption. Enhanced insulin secretion can be beneficial in patients with type 2 diabetes, because it acts to lower blood glucose, but in patients who are insulin sensitive, either because they were lean at the time of surgery for gastric cancer or because they lost a lot of weight after bariatric surgery, the high insulin levels can be sufficient to drive hypoglycemia. Mimicking the effects of bariatric surgery in obese and/or diabetic patients and reducing enteroendocrine activity in insulin-sensitive patients experiencing post-surgical hypoglycemia are both promising areas for developing new and effective treatments in the metabolic field.

## STAR★Methods

### Key Resources Table

**Table:** 

REAGENT or RESOURCE	SOURCE	IDENTIFIER
**Antibodies**		

rabbit anti CHGA	Abcam	Cat#Ab15160; RRID: AB_301704
rabbit anti SCG2	Abcam	Cat#Ab12241; RRID: AB_298964
donkey anti rabbit Alexa 647	Life technologies	Cat#A31573; RRID: AB_2536183
PE-coupled antibody anti-CD45 (EM-05)	ThermoFisher Scientifics	Cat#MA110233; RRID: AB_11153376
Anti GLP1R blocking antibody	Medimmune, Biggs et al., 2018	GLP1R0017
Ig control antibody	Medimmune	NIP228

**Chemicals, Peptides, and Recombinant Proteins**		

Exendin 9-39	Bachem AG	Cat#H8740.0500
Glucose	Sigma	Cat#G7528
70kDa FITC dextran	Sigma	Cat#FD70S
Acetonitrile	Pierce	Cat#51101
tryspin EDTA 0.25%	Life technologies	Cat#25200072
DNase1	VWR	Cat#A3778.0050
Y27632	Tocris	Cat#1254/10
DAPI	Sigma	Cat#D9542
Draq5	eBioscience Fisher Scientific	Cat#15530617
RNAsin plus RNase inhibitor	Promega	Cat#N2611
Saponin	Sigma	Cat#47036
Guanidine HCl	Sigma	Cat#G3272
Lyzing Matrix D beads	MPbiomedicals	Cat#116540434
HLB Prime micro elution plate	Waters	Cat# 186008052
DTT	Sigma	Cat#43815
iodoacetamide	Sigma	Cat#I1149
EDTA solution 0.5M	Sigma	Cat#03690
collagenase XI	Sigma	Cat#C9407

**Critical Commercial Assays**		

RNEasy plus micro kit	QIAGEN	Cat#74034
Clontech SMARTer stranded total RNaseq v2 pico kit	Takara Bio	Cat#634412
RNEasy Minelute cleanup kit	QIAGEN	Cat#74204
Rat/mouse total GIP ELISA	Merck Millipore	Cat#EZRMGIP-55K
MSD prototype Mouse/Rat Total PYY assay	MesoScale Discovery	
Mouse/Rat Insulin assay	MesoScale Discovery	Cat# K152BZC-3
Human Total PYY assay	MesoScale Discovery	Cat# K151MPD1
Human Total GIP assay	MesoScale Discovery	Cat# K151RPD1
Human insulin assay	DiaSorin	Cat#310360
Total GLP1 assay	MesoScale Discovery	Cat# K150JVC
Glucagon assay	Mercodia	Cat# 10-1271-01

**Deposited Data**		

RNaseq data from different regions of the GI tract in mouse and from jejunum in human after bariatric surgery	GEO repository	GEO: GSE121490
peptidomics data from different region of the GI tract in the mouse or human jejunum after bariatric surgery	PRIDE / ProteomeXchange	PRIDE: PXD011455; PRIDE: PXD009796; PRIDE: PXD011498

**Experimental Models: Organisms/Strains**		

Mouse: Neurod1-cre/EYFP: C57BL6	in house breeding	Project license 70/7824

**Software and Algorithms**		

R 3.4.2		https://www.rstudio.com/products/rstudio
STAR v2.5.1	Dobin et al., 2013	https://github.com/alexdobin/STAR/releases
Deseq2	Love et al., 2014	https://bioconductor.org/packages/release/bioc/html/DESeq2.html
Peaks v8.5		http://www.bioinfor.com/peaks-studio/
XCalibur 4.1		https://www.thermofisher.com/order/catalog/product/OPTON-30487

**Other**		

Ensure plus chocolate	Abbott laboratories	Cat#353-3601
45% high fat diet	Research Diets	Cat#D12451
10% high fat diet	Research Diets	Cat#D12450H

### Contact for Reagent and Resource Sharing

Further information and requests for resources and reagents should be directed to and will be fulfilled by the Lead Contact, Fiona Gribble (fmg23@cam.ca.uk).

### Experimental Model and Subject Details

#### Human Exendin 9-39 study

All human studies were conducted in accordance with the principles of the Declaration of Helsinki and following NHS HRA and REC approval (refs: 16/EE/0338; 16/EE/0545), and registered on clinicaltrials.gov (NCT02836353; NCT02971631). Post-gastrectomy participants (age 39.2 ± 8.2 [mean ± SD], 1Female:4Male, BMI 22.1 ± 1.8) were recruited from previous research studies and clinical follow-up at Addenbrooke’s Hospital. All had clinical or biochemical evidence of post-prandial hypoglycaemia. All were screened for anemia, and hepatic and renal dysfunction prior to recruitment and gave written consent following provision of a detailed information leaflet and discussion with the research team. The study required two overnight stays on the Translational Research Facility (TRF); four of five participants consolidated this to one two-night stay with study interventions on consecutive days (previous studies using Exendin 9-39 have demonstrated no ongoing effect of the agent after 12 hours).(Calabria et al., 2016)

Participants were admitted at 5pm and provided with a weight-standardized meal, following which they were permitted only water prior to the study the next day. A Dexcom G4 continuous glucose monitor was sited in the evening to allow it to stabilize and calibrate prior to study interventions. The following morning, participants were woken at 07:00 to allow study interventions to commence at 07:30.

GMP grade lyophilised synthetic Exendin 9-39 was purchased from Bachem AG (Switzerland), stored at −20°C and supplied to the TRF through the pharmacy supply chain of Addenbrooke’s Hospital. Exendin 9-39 or placebo infusion was prepared on the morning of the infusion by the nursing staff on the TRF and infused into a venous cannula. Participant and investigator were blinded to the infusion contents. Infusions were prepared in low-protein absorbing bags (Macoflex N) in 1% human albumin solution in normal saline and infused through low-protein absorbing tubing to reduce peptide adsorption. Placebo infusion was identical to the experimental infusion with the exclusion of only Exendin 9-39.

One cannula was sited in each ante-cubital fossa of the participant (one for infusion, one for blood sampling). Following collection of baseline bloods and symptom scores, Exendin 9-39 was given as a bolus (7500pmol/kg) over 4 minutes followed immediately by infusion at 500pmol/kg/minute(Craig et al., 2017), starting at T-40 minutes. Placebo bolus and infusion were at the same rate as for Exendin 9-39. Blood samples and visual analog scores (VAS) were collected prior to administration of a 50 g OGTT (at T0) and then every 15 minutes for two hours. Blood was collected into EDTA and LiHep tubes, placed immediately on wet ice, centrifuged at 3500 g for 10 minutes at 4°C, and the plasma separated and snap frozen in 500μl aliquots on dry ice within 30 minutes. VAS were collected by marking a 10cm line between the statements “Not at all” and “Extremely,” following the questions: “How hungry are you right now”? and “How full are you right now”?.

Initial power calculations suggested 13 participants would be required to reach significance on the primary outcome measure (nadir blood glucose concentration), however following a change in production policy at Bachem it was not possible to source a second batch of Exendin 9-39 at GMP grade and so the study was restricted to five participants.

#### Human transcriptomics and peptidomics study

Participants undergoing total gastrectomy (for the treatment or prevention of gastric cancer) with Roux-en-Y reconstruction consented to collection of a small cuff of jejunum from the apex of the alimentary limb of the reconstruction (i.e., just distal to the esophago-jejunal anastomosis) during surgery.

Participants recruited for endoscopy were either undergoing a clinically indicated procedure, or consented to a specific research endoscopy for tissue collection. For RNaseq, ten biopsies were collected from the apex of the alimentary limb of the Roux-en-Y reconstruction, for peptidomics two biopsies were collected from the apex of the alimentary limb. Different participants were recruited for RNaseq and peptidomics to reduce the biopsy burden on each participant. For RNaseq, tissue was used from 3 pre-operative (age 48 ± 24, all male) and 4 post-operative participants (age 48 ± 17, 3Male:1Female), and for peptidomics, from 7 pre-operative (age 69 ± 15, all male) and 4 post-operative participants (age 54 ± 12, 1Male:3Female). All tissue samples were immediately placed in L-15 media on ice and transferred to the laboratory for processing within 20 minutes.

#### Mouse VSG surgery

All animal work was performed under the UK Home office project license 70/7824 conforming to the according regulations (Animals Act Regulations SI 2012/3039).

Standard chow-fed lean male mice on a C57BL6 background aged ∼20 weeks and weighing 31.5 ± 3g were switched to liquid diet (Ensure plus chocolate, Abbott laboratories) and single housed 3-4 days before surgery. Surgery was performed as described(McGavigan et al., 2017). Briefly, mice were anaesthetised using 2%–4% isoflurane, injected with analgesic (Metacam, 2mg/kg, sc), the peritoneal cavity was opened and stomach isolated. For VSG operated mice, 70%–80% in volume of the total stomach was excised. The remaining stomach pouch was closed with 6-0 suture and returned to the abdominal cavity. For sham-operated mice, a continuous suture was placed along the line of transection for VSG operated mice but without applying constraint on the stomach. All animals were given analgesic for 2 days and antibiotics (Baytril, 20mg/kg, sc) for 7 days after surgery and monitored daily for signs of post-operative complications. Mice that presented evidence of infection or weight loss of > 25% of their weight on the day of the surgery were excluded from the experiment and humanely culled.

Mice were kept singly housed on cage liners and with standard enrichment on a 12h:12h light cycle. Food intake and body weight were measured 3 times a week. The sham-operated weight matched group received food twice daily to match their body weight to the VSG group. VSG operated mice also received 0.1mg/kg B12 (cyanocobalamin) weekly by subcutaneous injection.

Mice were kept on liquid diet for one week before being transitioned back to standard chow in the first experiment. For the GLP1R blockade study, mice were kept on the liquid diet for 4 weeks after surgery then 4 weeks on a high fat diet (45% energy from fat, D12451, Research Diets) followed by 2 weeks of control low fat diet (10% energy from fat, D12450H, Research Diets). All food transitions were done by presenting the new food to the animals one day before removing the previous food.

For the GLP1R blockade, mice were subcutaneously injected with 19.2mg/kg of GLP1R0017 blocking antibody(Biggs et al., 2018) or control antibody (NIP228, MedImmune) the day before surgery and then weekly for the first 5 injections and then intraperitoneally. Blood samples were collected just before the antibody injection every two weeks and at the end of the OGTTs to validate the presence of the antibody in the circulation.

### Method Details

#### Mouse OGTT, blood collection and Intestinal transit

In the first experiment, 4 weeks after surgery, mice were fasted overnight (16h) and then gavaged with 1g/kg glucose, and tail blood glucose levels were measured before and 5, 15, 30, 45, 60, 90 and 120 min after the gavage using a glucometer (Alpha Trak 2 pet blood glucose meter) and plasma samples for insulin and GLP-1 were collected at 0, 5 and 15min. The same protocol was used for the mice treated with the GLP1R blocking antibody, except that OGTTs were performed 2, 4 and 10 weeks after the surgery when on liquid and LFD respectively.

In the first experiment, 6 weeks after surgery, mice were fasted overnight and then received a 3g/kg glucose challenge. Tail blood samples were taken before and 5 minutes after the glucose challenge. At ∼15 minutes after gavage, terminal blood was collected by cardiac puncture and tissues harvested for the RNaseq analysis. Mice used for the peptidomics analysis were fasted overnight and terminal blood was collected by cardiac puncture after CO_2_ asphyxiation.

For the GLP1R blocking study, at 12 weeks after surgery, mice were fasted for 8h and gavaged with 100 μL Ensure with 0.5mg of 70kDa FITC-dextran (Sigma). Mice were killed by CO_2_ 5 minutes after the gavage, and terminal blood collected by cardiac puncture. Intestinal tissue was harvested and divided into 12 regions, 1 for the stomach / stomach remnant, 8 for small intestine, one for caecum and 2 for colon and rectum, numbered from 1 for the stomach to 12 for the rectum. Each region was opened and intestinal content washed in 1mL PBS or 3 mL of PBS for the stomach and caecum regions. Fluorescence of the intestinal content was measured using a Tecan M1000 Pro Plate Reader. Intestinal transit score was calculated as the geometric center of the fluorescence fraction per region(Miller et al., 1981). Plasma gut peptide levels were correlated to the intestinal transit score using a Pearson correlation.

Tail blood was collected in heparin-coated capillaries and terminal blood in EDTA coated tubes (Sarstedt); plasma was separated by centrifugation and immediately frozen and stored at −80°C until analysis. Hormone concentrations were measured using the assays detailed in the Key Resources Table.

#### Human plasma analysis

Human blood samples were collected into EDTA tubes for LC-MS, into lithium heparin tubes for measurement of insulin and glucose, and into EDTA tubes treated with DPP4 inhibitor (EMD Millipore) and aprotinin for glucagon, GLP-1, GIP and PYY. Samples were immediately placed on ice and centrifuged for 10 minutes at 3500 g at 4°C. Hormone concentrations were measured using the assays detailed in the Key Resources Table. Glucagon was measured using a modified version of the Mercodia sandwich immunoassay kit with additional wash steps to reduce cross-reactivity with other proglucagon species (Roberts et al., 2018b). To measure Exendin-9, a plasma calibration line of Exendin-9 was generated from 0.1 to 10.0 μg/mL and 20μL was extracted using 125μL of 80% ACN with 0.1% formic acid (FA). Supernatant (50 μL) was transferred to a Lo-bind plate and 450 μL of 0.1% FA in water added. LC-MS analysis was performed on a Waters TQ-XS mass spectrometer with a Waters H-Class UPLC system. Sample (5μL) was injected onto a Waters HSS T3 50x2.1mm column with a starting condition of 75% A (0.1% FA in water) and 25% B (0.1% FA in ACN) raising to 65%B over 3 minutes. Exendin 9 was detected using an SRM transition of 843.1 to 396.01 m/z. LC-MS data was processed using the TargetLynx XS software package (Waters).

#### Mouse EEC cell sorting and RNaseq

EECs were purified from Neurod1-cre / EYFP mice on chow diet. After the terminal glucose challenge, top 5cm and bottom 15cm of the small intestine, and large intestine were harvested in L-15 media and kept on ice until processing. Tissue segments were washed with ice-cold PBS and the muscle layer removed before being incubated at room temperature in PBS containing 15mM (30mM for the colon) EDTA and 1mM DTT for 7 min. Tissue was then transferred to a tube containing PBS with Y27632 Rock inhibitor (5 μM), shaken for 30 s, and the remaining tissue returned to the EDTA/DTT. Incubation and shaking steps were repeated 5 times. Villi and crypts in the PBS/Rock inhibitor solution were collected by centrifugation, incubated for 5 minutes with 0.25% Trypsin EDTA (Sigma) with 0.1mg/mL DNase1 at 37°C, recentrifuged, then completely dispersed into single cells in HBSS (H9394, Sigma) containing 10% FBS and Y27632, by trituration and filtration through a 50 μm filter. Cells were stained for 1h at 4°C with a PE-coupled antibody anti-CD45 (EM-05, ThermoFisher Scientifics) used at 1/500 then stained for 5min with DAPI 1 μg/mL in HBSS. Cells were rinsed twice and DRAQ5 (5 μM, Biolegend) was added. Cells were sorted on a FACSJAZZ (BD Bioscience) at the Cambridge NIHR BRC Cell Phenotyping Hub based on their size and scatter, excluding debris (DRAQ5 negative), dead cells (DAPI positive) and immune cells (CD45 positive) to collect 1,500 – 20,000 EECs (EYFP positive). Cells were directly sorted in RLT+ buffer with 1% β-mercaptoethanol and RNA was extracted using a RNeasy microplus kit (QIAGEN). Concentration and RNA quality were assessed using an Agilent 2100 Bioanalyser. All samples had a RIN value > 5 except for one sample which clustered with all others after analysis.

Libraries for sequencing from 2ng of RNA from each sample were generated using the SMARTer stranded total RNaseq v2 pico kit (Takara Bio) and libraries were pooled together and single-end 50 bases sequenced on an Illumina Hiseq 4000 at the CRUK Cambridge Institute Genomics Core.

#### Human EEC sorting and RNaseq

FACS and RNA extraction from fixed human cells followed a modified version of the MARIS protocol (Hrvatin et al., 2014, Roberts et al., 2018a). Intestine was rinsed in cold PBS and the muscular coat removed. Diced mucosa was digested twice in 0.1% w/v collagenase XI (Sigma-Aldrich) in HBSS (Sigma-Aldrich) for 30 minutes each time at 37oC, shaking vigorously every 10 minutes. Supernatants were triturated, passed through a 50μm filter and centrifuged at 300 g. Pellets were resuspended in PBS and fixed in 4% w/v paraformaldehyde (PFA) at 4°C for 20 minutes. PFA-fixed cells were washed twice in nuclease free 1% w/v bovine serum albumin (BSA) in PBS, and if a fluorescence assisted cell sorting (FACS) facility was not immediately available, were suspended in 1% w/v BSA and 4% v/v RNAsin plus RNase inhibitor (Promega, WI, USA) in PBS at 4°C overnight.

Cells were permeabilised by the addition of 0.1% w/v Saponin (Sigma-Aldrich) to solutions in all steps from this point until after the first wash post-secondary antibody staining. Primary antibody staining used 2% v/v rabbit anti-CHGA (Abcam, Cambridge, UK; Ab15160) and 0.25% v/v rabbit anti-SCG2 (Abcam, Ab12241) and was for one hour in 4% v/v RNAsin, 1% w/v BSA and at 4°C. Cells were then washed twice in 1% w/v BSA, 1% v/v RNAsin, and secondary antibody staining was for 30 minutes in 4% v/v RNAsin, 1% w/v BSA and 0.2% v/v donkey anti-rabbit Alexa 647 in PBS at 4°C. Cells were washed twice then suspended in 4% v/v RNAsin, 1% w/v BSA in PBS on ice for FACS.

Cell populations were sorted on a BD FACS ARIA III in the Cambridge NIHR BRC cell phenotyping hub or at Institut Cochin, Paris. Single cells positive for Alexa 647 were classified as EECs. At least 5000 cells were collected for each positive population. Twenty thousand negative cells were collected as the negative (i.e., non-enteroendocrine) cell population. Cells were sorted into 2% v/v RNAsin in PBS at 4°C.

RNA was extracted using the Ambion Recoverall Total nucleic acid isolation kit for FFPE (Ambion, CA, USA) with modifications to the protocol as below. The FACS sorted cell suspension was centrifuged at 3000 g for 5 minutes at 4°C and the pellet resuspended in 200μl digestion buffer with 4μl protease and incubated at 50°C for 3 hours. The solution was then stored at −70°C for at least 12 hours prior to further extraction. After thawing, RNA was extracted using the manufacturer’s protocol (including a DNase step) with the exception of performing 2x 60μl elutions from the filter column in the final step.

The RNA solution was concentrated using a RNEasy Minelute cleanup kit (QIAGEN, Hilden, Germany). RNA aliquots were diluted to 200μl with nuclease free water. The standard manufacturer’s protocol was followed with the exception that 700μl, not 500μl, of 100% ethanol was added to the solution in step two, to generate optimum binding conditions for the PFA fragmented RNA. RNA concentration and quality was analyzed using an Agilent 2100 Bioanalyser (Agilent, CA, USA).

cDNA libraries were created using the Clontech SMARTer Stranded Total RNA-Seq Kit – Pico Input Mammalian v2 (Takara Bio, USA). RNA input quantity was 5ng and the non-fragmentation protocol was used. The standard manufacturer’s protocol was followed with the exception that 175μl of AMPure beads were used for the final bead purification to ensure recovery of the small fragments of RNA arising from PFA fixation. Sixteen PCR cycles were used for amplification. 50 base single-end sequencing was performed using an Illumina HiSEQ 4000 at the CRUK Cambridge Institute Genomics

#### Peptidomics of intestinal tissue

Intestinal tissue from fasted mice was harvested and sections of 10-20mg were collected every 5cm along the GI tract (from 7 positions in the small intestine and 3 positions in the large intestine), the stomach remnant and different part from the stomach of the sham operated mice (lesser curvature, corresponding approximatively to the position of the sample collected in VSG operated mice, corpus, antrum and fundus) from mice following glucose challenge, and pancreas samples from the mice used for the intestinal transit challenge (ie after an Ensure challenge), as well as human biopsies collected as previously described. Tissue samples were directly placed in 250 μL 6M guanidine HCl and homogenized using Lysing Matrix D beads (MPbio). In a Lobind protein tube (Eppendorf), 800 μL ACN 80% (v/v in water) was added to the samples to precipitate proteins and centrifuged 12,000 g centrifugation at 4C for 5min. The aqueous (lower) phase was recovered and dried using a centrifugal vacuum concentrator at room temperature. Samples were then resuspended in 500 μL 0.1% formic acid in water and spiked with internal standard, and peptides were purified by solid phase extraction (Oasis prime HLB, Waters) and eluted in 60% methanol with 10% acetic acid and 30% H_2_O. Samples were dried using a nitrogen flux at 40°C (SPE Dry evaporator system, Biotage) and resuspended in 40 μL 10mM DTT in 50mM ammonium bicarbonate for 1h at 60°C. 10 μL of 100mM iodoacetamide was added and samples incubated for 30min at room temperature in the dark for reduction alkylation. Finally, 60 μL 0.1% formic acid in water was added and 10 μL of the samples was injected into a high flow (for the small and large intestine samples) or a nano flow (stomach and pancreas samples) on a Thermo Fisher Ultimate 3000 nano-LC system coupled to a Q Exactive Plus Orbitrap mass spectrometer (ThermoScientific) as described previously (Kay et al., 2017, Roberts et al., 2018a). A full scan of 400-1600 m/z was performed and the top 10 ions of each spectrum were selected for fragmentation and MS/MS analysis.

### Quantification and Statistical Analysis

Data were analyzed and represented using R (v3.4.2) and statistical analysis is described in figure legends accordingly to the data analyzed as well as the n numbers. Data are represented by median and individual values when possible except when readability required to present mean ± sd. In the human studies, we did not perform subgroup-analyses by age or gender, due to the low n-numbers.

#### Mouse and Human RNasequencing data analysis

Sequenced reads were demultiplexed, quality checked and were aligned on the mouse (GRCm38) or human (GRCh38) genome and raw counts generated using STAR v2.5.1(Dobin et al., 2013) using the GRCm38.91 or GRCh38.93 annotations. Gene expression was analyzed using DESeq2 using the operation group in interaction with the region (mouse only) in the model for normalization, log2 fold change estimation using the lfcShrink function and differential gene expression. Results from the human Deseq2 analysis were then subset to only the genes that are enriched in human EECs as assessed previously(Roberts et al., 2018a).

#### Mouse and Human peptidomics data analysis

LC-MS/MS data were analyzed using Peaks v8.5 software using the *Mus musculus* or the *Homo sapiens* Swissprot database (downloaded 26/10/2017), searching for intact peptides of less than 65 amino acid length, with fixed cysteine carbamidomethylation, while methionine oxidation, N-terminal pyro-glutamate, N-terminal acetylation and C-terminal amidation modifications were variable. Relative levels of peptides were assigned by integrating peptide peak areas specifically designated by their *m/z* and retention time using the QuanBrowser module in Xcalibur (ThermoFisher) and normalized using the spiked internal standards and the tissue weight.

#### Insulin rate analysis

Insulin secretory rate (ISR) was calculated using a two-compartment C-peptide deconvolution model, accounting for age, gender and body surface area, using the ISEC program. Linear mixed effects models were generated as described using the package lme4 in RStudio, with the lmerTest modification. Model fit and normality were tested by plotting the residuals against the fitted value (check for heteroscedasticity), QQ plots for normality of residuals, plots of fitted versus predicted variables to check accuracy of model, and residual-leverage plots to check for highly leveraged data points.

### Data and Software Availability

Transcriptomics data have been deposited on the GEO repository under the GEO: GSE121490 reference series (https://www.ncbi.nlm.nih.gov/geo/query/acc.cgi?acc=GSE121490), divided in two subseries separating human (GSE121486) and mouse data(GSE121489).

Peptidomics data have been deposited on the ProteomeXchange Consortium via the PRIDE repository. Mouse data is available with the project accessions PRIDE: PXD011455 and PRIDE: PXD009796, and human data with the project accessions PRIDE: PXD011498.

## Supplemental Information

Document S1. Figures S1–S4

Document S2. Article plus Supplemental Information
