# Supplementary material for: Important Role of the GLP-1 Axis for Glucose Homeostasis after Bariatric Surgery
Source: Cell Rep. 2019 Feb 5;26(6):1399–1408.e6. doi: 10.1016/j.celrep.2019.01.047 (PMC6367566; doi:10.1016/j.celrep.2019.01.047)
Supplement: Document S1. Figures S1–S4 [file mmc1.pdf]

Cell Reports, Volume *26*

Supplemental Information

Important Role of the GLP-1 Axis for Glucose Homeostasis after Bariatric Surgery

Pierre Larraufie, Geoffrey P. Roberts, Anne K. McGavigan, Richard G. Kay, Joyce Li, Andrew Leiter, Audrey Melvin, Emma K. Biggs, Peter Ravn, Kathleen Davy, David C. Hornigold, Giles S.H. Yeo, Richard H. Hardwick, Frank Reimann, and Fiona M. Gribble

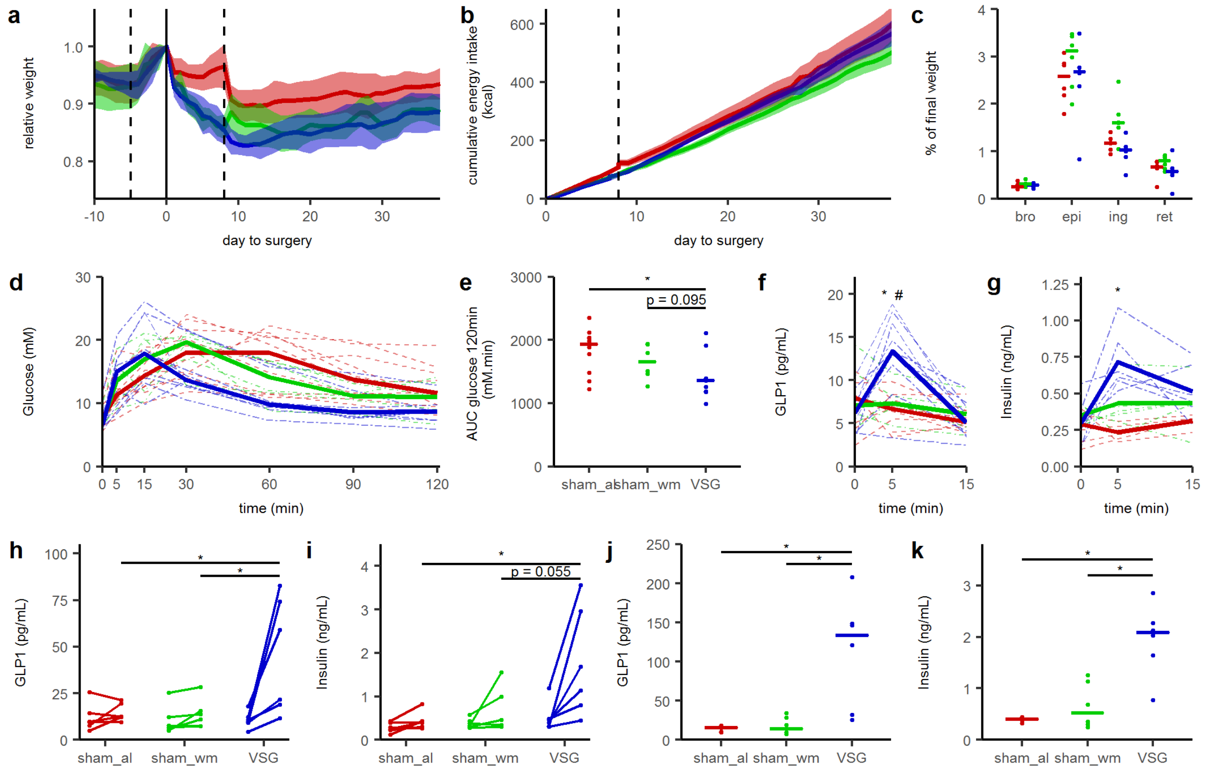

Figure S1: Effect of Vertical sleeve gastrectomy on lean mice (Related to Fig 2)

Lean mice had either a vertical sleeve gastrectomy (VSG) or a sham control operation involving placement of a gastric purse string suture but no gastric excision (McGavigan et al., 2017). An additional control sham group was weight-matched to the VSG group by calorie restriction. All mice received a liquid diet beginning 4 days before surgery and displayed accelerated weight gain during this period. As expected, the VSG group and weight-matched sham groups exhibited more weight loss during the first week after surgery than sham controls, although when transferred from liquid to chow diet after 7 days, there was some slight catch up of the VSG group (a). Weight changes were associated with similar changes in cumulative energy intake (b). VSG operated mice (n = 9, dark blue), sham operated mice (n=10, red) and sham operated mice weight matched to the VSG group (n =6, green). Vertical dashed line represents the change of diet from liquid diet to standard chow diet. Data are mean ± sd. c: relative adipose tissue weight to total mouse weight from different tissue pads. Bro: brown adipose tissue, Epi: epidydimal, Ing: Inguinal, Ret: retroperitoneal fat.

d: blood glucose levels after a 1g/kg oral glucose gavage, 4 weeks after surgery; dashed lines are individual mice and solid lines are the median per condition. e: Area under the curve for glucose response over 120min to a 1g/kg OGTT, for data shown in d. f, g: Plasma total GLP-1 and insulin levels 0, 5 and 15 min after a 1g/kg OGTT. h, i: Plasma total GLP-1 and insulin levels 0 and 5 min after a 3g/kg OGTT. j, k: Plasma total GLP-1 and insulin levels in terminal blood 10 min after a 3g/kg OGTT. As these terminal samples were taken by cardiac puncture after CO_2 anaesthesia, they are depicted separately, as we cannot be certain that the absolute hormone concentrations are comparable with those measured from tail-bleeds of the non-anaesthetised mice at earlier time points.

Data are represented as individual points with median, * indicates a statistical difference with a cut-off of □ = 0.05 assessed using a Dunn's test after validation that all groups did not all come from a same population with $p<0.05$ by a Kruskal-Wallis test.

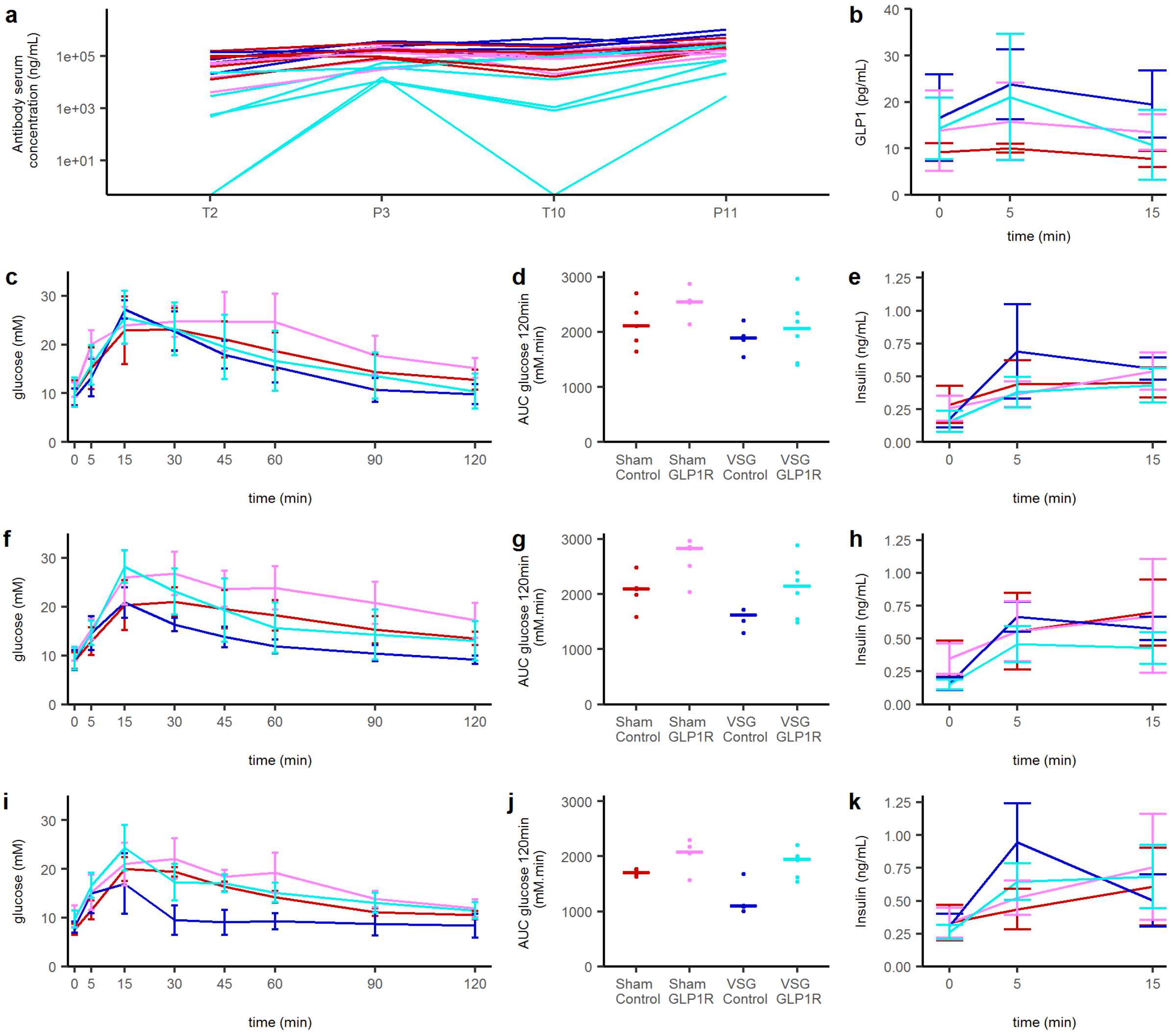

Figure S2: Additional data for the murine GLP1R antibody study (Related to Fig 2)

VSG operated mice were treated with control antibody (n=4, dark blue) or GLP1R antibody (n = 6, light blue) and sham operated mice treated with control antibody (n=5, dark red) or GLP1R antibody (pink).

a: Peak and trough antibody concentrations, 2 weeks and 10 weeks after the start of the antibody injections. b: GLP-1 concentration in response to a 1g/kg glucose challenge 10 weeks after surgery.

c, f, i: Glucose responses to a 1g/kg glucose 2 (c), 4 (f) and 10 (i) weeks after surgery. Values are mean ± sd. d, g, j: Area under the curve over 120 min for blood glucose responses shown in c, f and i. Individual mice and medians are presented. e, h, k: Plasma insulin levels in response to 1g/kg glucose challenge 2 (e), 4 (h) and 10 (k) weeks after surgery, data are mean ± sd. Data in c-k represent the raw results that were combined to form the plots shown in Figure 3.

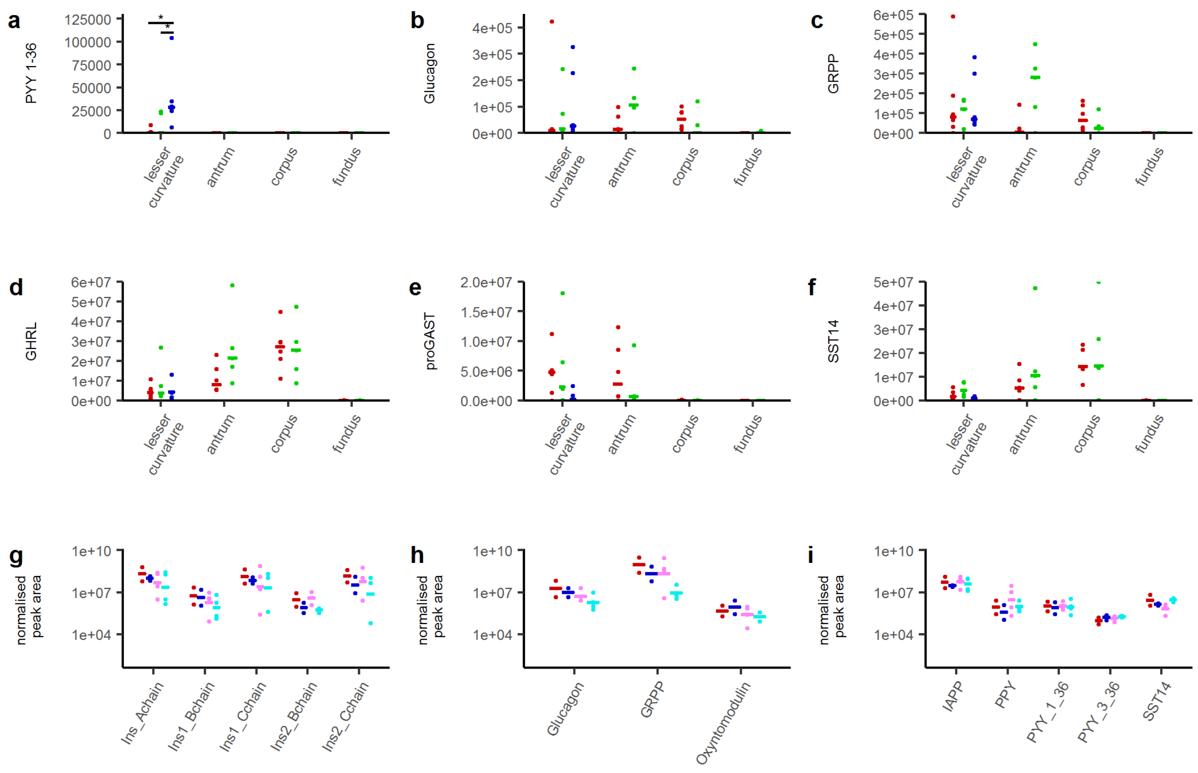

Figure S3: Peptide quantification in stomach and pancreas (Related to Fig 3)

a-f, Quantification of PYY1-36 (a), Glucagon (b), GRPP (c), Ghrelin (d), Nterminal part of proGastrin (e) and SST14 (f) in different regions of the stomach or the stomach remnant, considered to be equivalent to the stomach lesser curvature, represented as median and individual samples from sham fed ad libitum (red) or weight-matched (green) and VSG (blue) -operated mice.

g-h, Quantification of individual peptides related to insulin (a), proglucagon (b) and others (c) in pancreas homogenates, represented as median and individual samples from sham (red) and VSG (blue) -operated mice treated with control (dark) or GLP1R (light) antibody. GLP-1(1-37) levels were low and were detectable in some but not all samples, so have not been included in the figure. Differences between groups were assessed in each tissue for each peptide using a Dunn test.

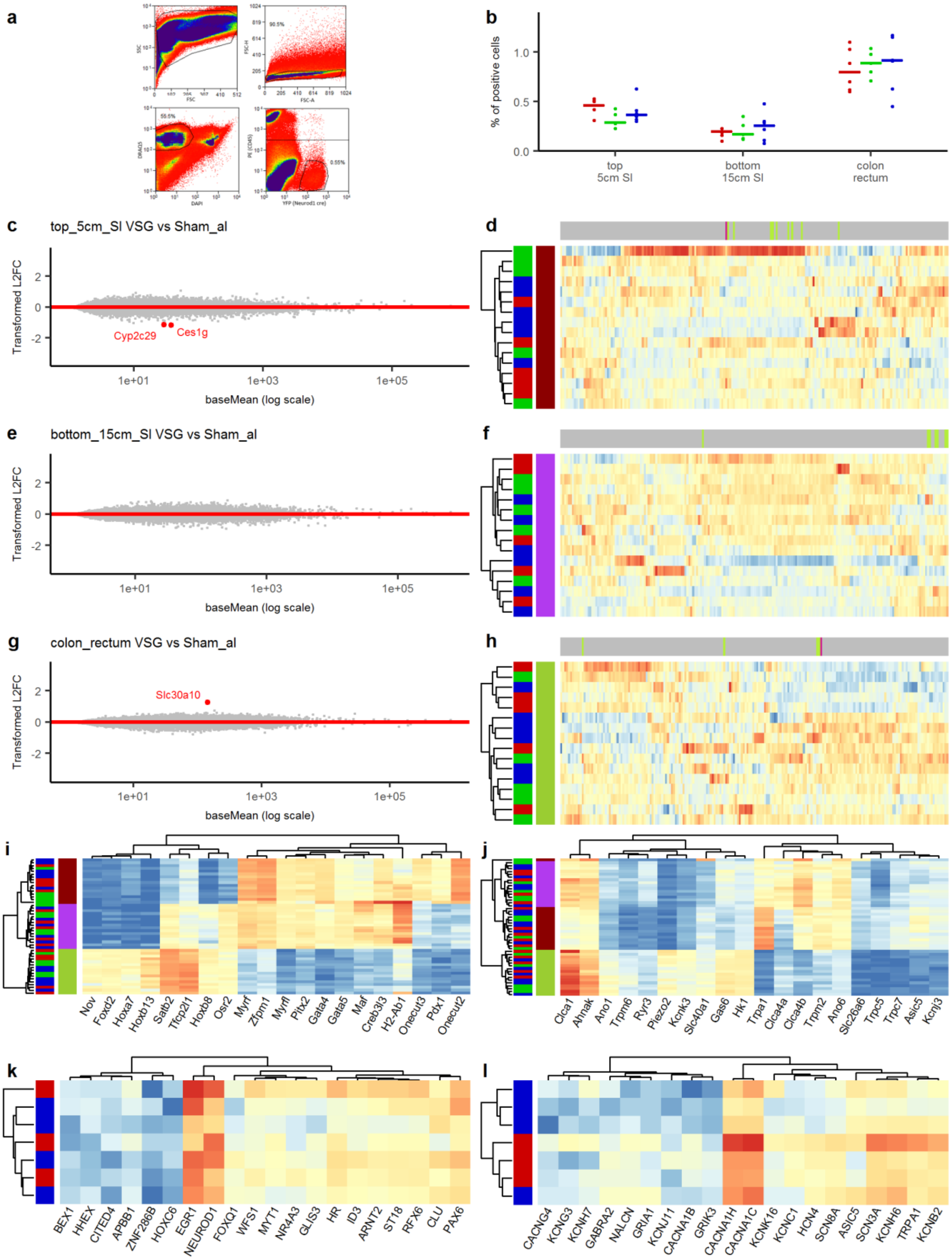

Figure S4: Additional data for transcriptomics of murine EECs (Related to Fig 4)

a: Representative sorting strategy for EECs, sorting cells based on their forward and side scatter, removing doublets, selecting live intact cells (Draq5 positive and DAPI negative) and eYFP positive cells, excluding immune CD45-PE positive cells. b: percentage of positive cells sorted in each tissue, data are median with individual samples for sham fed *ad libitum* (red), sham weight matched (green) and VSG (blue) -operated mice

c, e, g: MAplots of the VSG vs sham-*ad libitum* samples in duodenum (c), ileum (e) and colon (g) representing the log2 fold change between conditions and the mean normalised expression of each individual genes using DESeq2 models on all samples from one tissue. Genes which are differently expressed are annotated in red (padj<0.05).

d, f, h: Heatmaps representing the log2 normalised expression of the top 100 variable genes across all samples in duodenum (d), ileum (f) and colon (h). Variance was calculated excluding one sham weight-matched duodenal sample that was an outlier. Differently expressed genes as determined by Deseq2 are highlighted, red for differently expressed in VSG vs sham ad-libitum fed, and VSG vs sham weight-matched, pink for differently expressed only in VSG vs sham ad-libitum and green for only differently expressed in VSG vs sham weight-matched. Samples and genes are clustered by Euclidean distance with scaling per gene.

i,j: Transcriptomic analysis of murine NeuroD1-positive EECs from different regions of the gut. Heatmaps represent the log2 normalised expression of top 25 variable genes across all samples that are annotated transcription factors (i), and ion channels (j). Samples and genes are clustered by Euclidean distance without scaling.

k,l: Transcriptomic analysis of human jejunal EECs. Heatmaps represent the log2 normalised expression of top variable EEC-enriched genes annotated as encoding transcription factors (k) and ion channels (l). Samples and genes are clustered by Euclidean distance without scaling.
